# Effects of swimming exercise on high-fat diet-induced low bone mineral density and trabecular bone microstructure in rats

**DOI:** 10.20463/jenb.2016.0063

**Published:** 2017-06-30

**Authors:** Yun-Seok Kang, Sang-Hyun Kim, Jae-Cheol Kim

**Affiliations:** 1. Department of Sports Science, Chonbuk National University, Jeonju Republic of Korea

**Keywords:** Swimming exercise, Bone mineral density, Microstructure, Trabecular bone, High-fat diet

## Abstract

**[Purpose]:**

This study aimed to investigate the effect of swimming exercise on high-fat diet-induced low bone mineral density (BMD) and trabecular bone microstructure in rats.

**[Methods]:**

Eight-week-old male Sprague– Dawley (SD) rats were divided into a normal diet group (n = 9) and a high-fat diet group (n = 15). Three rats in each group were sacrificed after 8 weeks of high-fat diet to evaluate the association between high-fat diet and bone health. The other 18 rats were reassigned to 3 groups (normal diet control, NC; high-fat diet control, HC; high-fat diet + Exercise, HEx) for up to another 8 weeks. Rats in the exercise group were trained for a swimming exercise program (1 h/day, 5 times/ week for 8 weeks). All rats were sacrificed 24 h after the last bout of exercise to analyze the BMD and trabecular bone microstructure in the femur and tibia, using micro-computed tomography.

**[Results]:**

First, the effect of high-fat diet on bone health was examined. It was observed that BMD, percent bone volume (BV/TV), and trabecular number (Tb.N) of the femur and tibia were lower in rats in the high-fat diet group than in those in the normal diet group (p < .05). In addition, BMD, BV/TV, and Tb.N of the femur and tibia were significantly increased in rats that underwent the 8-week swimming exercise program, compared to the corresponding values in rats in the HC group (p < .05).

**[Conclusion]:**

These results indicate that high-fat diets negatively affect bone health; however, these negative effects can be improved by exercises such as swimming.

## INTRODUCTION

Bones are active living tissues that are sensitive not only to external stimuli such as exercise but also to metabolic changes within the body, and actively undergo metabolism^26^. During bone metabolism, resorption by osteoclasts is balanced by formation by osteoblasts, to repair microdamage and replace old tissues with new ones, and to maintain bone health^23^. 

Bone metabolism is regulated by the differentiation of marrow mesenchymal stem cells, which can differentiate into osteoblasts and adipocytes^6^. From childhood to early adulthood, which is the period of increase in bone mass, the differentiation of marrow mesenchymal stem cells into osteoblasts increases owing to the effects of growth factors; however, during the period of a decrease in bone mass as a result of aging, differentiation into osteoblasts decreases, while differentiation into adipocytes increases; this phenomenon inhibits normal bone metabolism^18^. Osteopenia and osteoporosis are characterized by reduced bone mineral density, and can result from such metabolic changes in the bone^17^. Moreover, excessive accumulation of fat, as observed in obesity, induces the expression of various inflammatory cytokines, and promotes the differentiation of marrow mesenchymal stem cells and hematopoietic stem cells into osteoclasts to increase bone resorption and decrease bone mineral density, ultimately increasing the risk of osteoporosis and fractures^20^. Sawant *et al*. reported lower bone mineral density of the tibia and femur in rat models of high-fat diet-induced obesity than in normal rats^29^. This result indicates that high-fat diet-induced obesity has negative effects on bone mineral density. 

Studies have been performed to identify methods to inhibit reduction in bone mineral density and stabilize or improve the existing levels. Among these methods, exercise is reported to not only provide appropriate stimuli for enhancing bone health but also increase the expression of growth factors, while reducing that of inflammatory factors by inducing weight loss, thereby positively affecting bone metabolism and bone health^13, 26^. Weight-bearing exercises such as running on a treadmill have most commonly been used in research^12, 21, 27^. However, several recent studies suggest that swimming can also effectively improve bone mineral density^7, 14^. Ju *et al*. reported increased femoral density and trabecular size after 12 weeks of swimming exercise, and found that muscle contraction during swimming exercise can sufficiently improve bone mineral density^14^. However, with numerous suggestions that underwater exercises such as swimming, in which the load is reduced by buoyant force, hinder improvement in bone mineral density^1, 24^, further investigation is necessary to clarify the effects. Swimming exercise can be effective, as it not only improves metabolic disorders related to obesity by reducing the joint load and contributing to weight loss, but also positively affects bone health by improving bone mineral density. 

The present study aimed to investigate the effects of swimming exercise for 8 weeks on bone mineral density and microstructural changes in the tibia and femur of rats. 

## METHODS

### Animal models

Eight-week-old male Sprague–Dawley rats (n = 24) were obtained from Damul Science Inc. (Daejeon, Korea). The rats were raised in a plastic cage (2 rats/cage) at the laboratory animal center of J University at 23–25°C and 70–80% humidity, with diurnal lighting. 

### Methods

#### Dietary treatment

**Figure 1.**
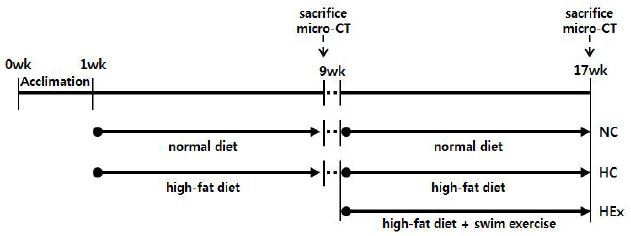
Experimental design. NC, normal diet control; HC, high-fat diet control; HEx, high-fat diet + swimming exercise.

The animals were acclimated to the environment for 1 week. The rats were randomly assigned to a normal diet group ([n = 9] that was fed a diet containing 3.5% fat), and a high-fat diet group ([n = 15] that was fed a diet containing 45% fat). The rats were fed *ad libitum* for 8 weeks (Table 1). Three rats were sacrificed from each group to analyze the reduction in bone mineral density due to the high-fat diet. After 8 weeks of dietary treatment, rats were assigned to normal diet (NC, n = 6), high-fat diet (HC, n = 6), and high-fat diet + swimming exercise (HEx, n = 6) groups. The experimental groups are described in <Fig. 1>. 

**Table 1:** Composition of experimental diet (mg)

	Normal diet	High-fat diet
Casein	200.0	245.0
L-Cystine	3.5	3.5
Corn Starch	397.5	85.0
Maltodextrin	35.0	115.0
Sucrose	100.0	200.0
Lard	20.0	195.0
Soybean Oil	70.0	30.0
Cellulose	50.0	58.0
Mineral Mix	30.5	43.0
Calcium Phosphate	3.5	3.4
Vitamin Mix	19.0	19.0
Choline Bitartrate	2.5	3.0
Red Food Color	-	0.1
Protein	20.5 %kcal	19.0 %kcal
Carbohydrate	76.0 %kcal	36.2 %kcal
Fat	3.5 %kcal	44.8 %kcal
Total	100 %kcal	100 %kcal

#### Exercise method

Rats were subjected to low-intensity free swimming exercise without a load, similar in intensity to running on a treadmill (13 m/min, grade 0%)^32, 33^. One week before the start of dietary treatment, rats underwent adaptation training (10–45 min/day) for 3 days in a water- filled (36°C ± 2°C) barrel (diameter 70 cm × height 70 cm). They were then subjected to swimming exercise (45–60 min/day, 5 times/week) for 8 weeks. The temperature of the water was maintained at 36°C ± 2°C, at which the core temperature is not affected^14^. 

#### Tissue collection

After 8 weeks of dietary treatment and exercise training, the rats were anesthetized with an intraperitoneal injection (1 mL/kg) of Zoletil (Virbac Laboratories, Paris, France), Rombun (Bayer Korea, Seoul, Korea), and saline solution mixed in a 2:1:2 ratio. Then, the tibias and femurs were separated and stored in a tube containing 4% formalin until the day of measurement^28^. 

### Measurement parameters and method of analysis

#### Micro-computed tomography (CT)

To analyze tibial and femoral bone mineral density and microstructure, micro-CT (SkyScan 1076; Bruker, Kontich, Belgium) was performed (voltage: 60kA, current: 167μA) through a 0.5-mm thick aluminum filter. The micro-CT images were reproduced in grayscale using Nercon Ver 1.3(SkyScan). The 2-dimensional (2D) images were reconstructed as 3D models by using CTAn and CTVox (SkyScan). A region of interest (ROI) between the growth plate of the femur or tibia to a point 0.5–4 mm away was analyzed using CTAn. Percent bone volume (BV/TV) of the ROI, which describes bone mineral density and microstructures, trabecular thickness (Tb.Th), trabecular number (Th.n), and trabecular separation (Tb. Sp) were measured. On CTVox, images of 2D cross-sectional planes and 3D structures of trabecular bone within the femurs and tibias were created. 

### Statistical analysis

The means and standard deviations of the collected data were calculated using SPSS Win 12.0. Weight differences between rats in the experimental groups after 8 weeks of a high-fat diet and swimming exercise were analyzed at different time points during the experiment using two-way repeated measures ANOVA. When interactions were observed between the main effects, oneway ANOVA was used to analyze the differences within and between groups at different time points. A t-test was used to determine the differences in bone mineral density between rats in the experimental groups after 8 weeks on a high-fat diet. One-way ANOVA was used to analyze the differences in the effects of swimming exercise after 8 weeks of a high-fat diet. The level of statistical significance was set at p < .05. 

## RESULTS

### Weight changes due to high-fat diet and swimming exercise

Weight changes in the rats are shown in Table 2. Significant differences after 8 weeks of dietary treatment and swimming exercise were found at different time points within (F = 2901.837) and between the groups (F = 186.486) (p < .05). A significant interaction was found between time point * groups (F = 33.428) (p < .05). A post-hoc test, with which weight changes at different time points were compared, showed significant changes in the weight during the diet period and the final weight relative to the starting weight in rats in all three groups (F = NC: 3,704.059, HC: 6,755.808, HEx: 2,849.430). Weight differences at different time points during the diet period were significantly higher in rats in the HC and HEx groups, which were assigned to a high-fat diet, than in those in the NC group, assigned to a normal diet (F = 106.378, p < .05). The final weight of rats in the HC group was significantly higher (F = 49.684, p < .05) than the final weight of rats in the NC and HEx groups. The final weights of rats in the HEx and NC groups were significantly lower than the final weights of rats in the HC group, indicating that 8 weeks of swimming exercise can decrease weight gained by a high-fat diet. 

**Table 3:** Multivariable hazard ratio (95% CI) for the risk of all-cause mortality according to physical activity levels in Korean adults.

Group	Start weight(g)	Diet period weight(g)	Final weight(g)	F-value		
				Group	Time	Group* Time
NC(n=6)	162 ± 4.1	422 ± 4.8a	490 ± 9.5b			
HC(n=6)	160 ± 3.5	471 ± 5.1a#	547 ± 8.2b#	186.485*	2901.837*	33.428*
HEx(n=6)	160 ± 4.4	474 ± 8.8a#	515 ± 11.1b			

Values expressed as mean±standard error(g).

* p<.05

a p<.05 vs. start weight in post hoc

b p<.05 vs. diet period weight in post hoc

# p<.05 vs. NC group in post hoc

### Changes in bone mineral density and morphology due to a high-fat diet

**Figure 2.**
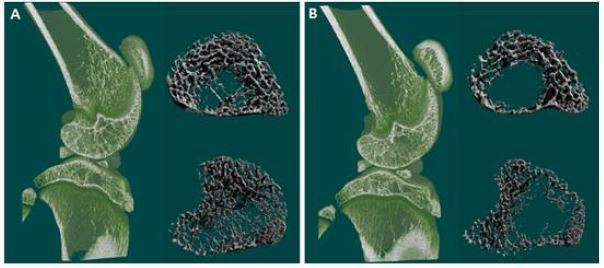
Typical 2- and 3-dimensional images of femur and tibia in normal diet(A) and high-fat diet(B) feeding rats, as visualized using micro-computed tomography.

**Figure 3.**
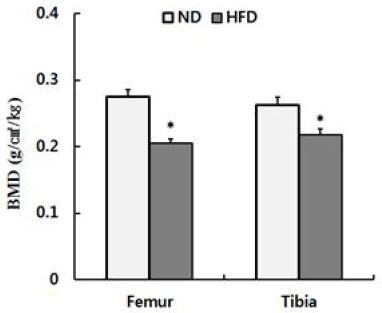
TMicro-computed tomography derived trabecular bone mineral density at the femur and tibia in normal diet(ND) and high-fat diet(HFD) feeding rats. *p<.05.

Morphological changes in the tibias and femurs during 8 weeks of dietary treatment are shown in Fig 2. Significant morphological changes (reduced trabecular area and number) were observed in the 2D and 3D images of the tibias and femurs of rats from the high-fat diet group relative to those of rats in the normal group. Rats in the high-fat diet group had significantly lower bone mineral densities of the tibias (F = .645) and femurs (F = .038) than those of rats in the normal group (p < .05) (Fig. 3). 

**Figure 4.**
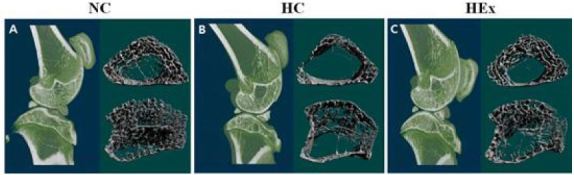
Typical 2- and 3-dimensional images of femur and tibia in normal diet control(A), high-fat diet control(B) and high-fat diet + swimming exercise(C) rats, as visualized using micro-computed tomography.

### Changes in bone mineral density due to swimming exercise after a high-fat diet

**Figure 5.**
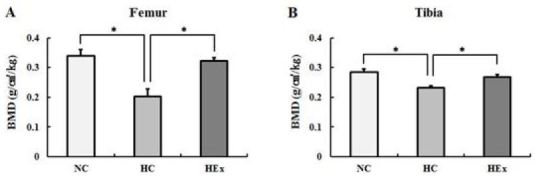
Micro-computed tomography derived trabecular bone mineral density at the femur(A) and tibia(B) in normal diet control(NC), highfat diet control(HC) and high-fat diet + swimming exercise(HEx) rats. *p<.05.

Bone mineral densities of the femurs (F = 43.551) and tibias (F = 20.843) measured after 8 weeks of swimming exercise were significantly reduced in rats in the HC group relative to those of rats in the NC group (p < .05). Similar results with regard to bone mineral density were observed between rats in the HEx group, which were subjected to swimming exercise, and those in the NC group (Fig. 5). 

### Changes in trabecular microstructure after swimming exercise following a high-fat diet

**Figure 6.**
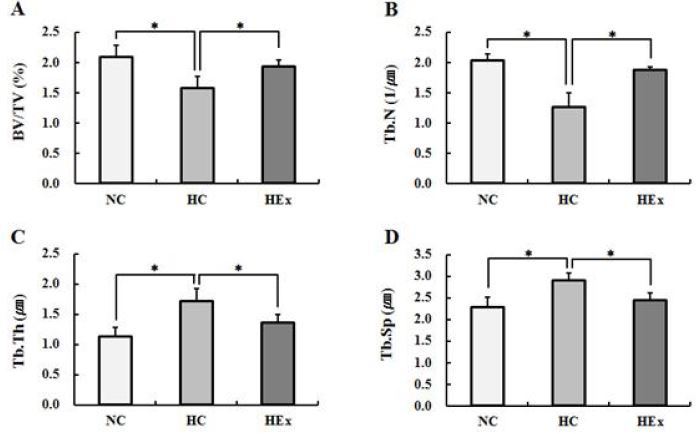
Micro-computed tomography derived trabecular bone parameters at the femur in normal diet control(NC), high-fat diet control(HC) and high-fat diet + swimming exercise(HEx) rats. (A) BV/TV : percent bone volume, (B) Tb.N : trabecular number, (C) Tb.Th : trabecular thickness, (D) Tb.Sp : trabecular separation. *p<.05.

**Figure 7.**
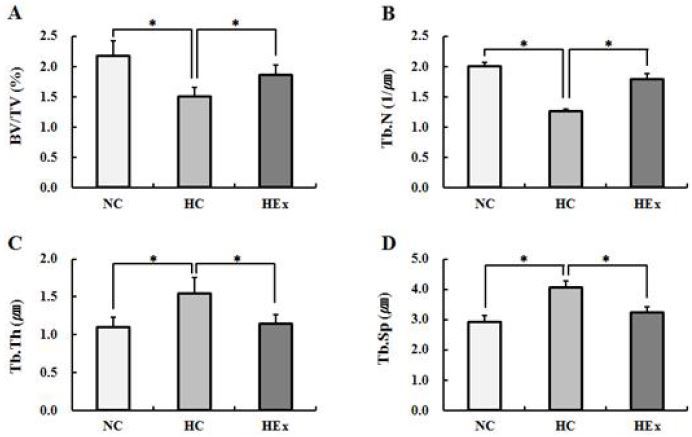
Micro-computed tomography derived trabecular bone parameters at the tibia in normal diet control(NC), high-fat diet control(HC) and high-fat diet + swimming exercise(HEx) rats. (A) BV/TV : percent bone volume, (B) Tb.N : trabecular number, (C) Tb.Th : trabecular thickness, (D) Tb.Sp : trabecular separation. *p<.05.

Changes in trabecular microstructure in femurs and tibias after 8 weeks of swimming exercise are shown in Fig. 6 and Fig. 7, respectively. BV/TV (F = 8.216) and Tb.N (F = 32.872) of the femurs were significantly reduced in rats in the HC group than in those in the NC group, significantly higher in rats in the HEx group than in the rats in the HC group, and similar to those of rats in the NC group, after 8 weeks of swimming exercise (p < .05). 

Tb.Th (F = 9.840) and Tb.Sp (F = 8.110) of femurs were significantly increased in rats in the HC group compared to those in rats in the NC group, significantly decreased in rats in the HEx group compared to those in rats in the HC group, and similar to those of rats in the NC group, after 8 weeks of swimming exercise (p < .05). 

Significant differences in BV/TV (F = 9.383) and Tb.N (F = 57.102) of tibias were found between rats in the HC group and those in the NC and HEx groups. Significant differences of Tb.Th (F = 14.200) and Tb.Sp (F = 23.733) were found between rats in the HC group and those in the NC and HEx groups (p < .05). 

## DISCUSSION

Important factors associated with bone health and strength include not only bone mineral density but also characteristics of bone microstructure^15, 16^. Micro-CT is frequently used to assess bone microstructure, and is an effective method of measurement that requires lesser time than that required by histomorphometry; the use of micro-CT also prevents morphological changes in the sample during analysis^31, 34^. It provides structural information by enabling the quantification of bone area, bone volume fraction, and number, gap distance, and thickness of trabecular, as well as 3D reconstruction of bone microstructures^9^. Bone mineral density and structure of cancellous bone rather than cortical bone are known to determine bone strength. The proportion of cancellous bone and the number of trabecular are decreased throughout the bone area in patients with osteopenia or osteoporosis, while the gaps between trabecular are increased owing to the reduced microstructure in cancellous bone, with increased average trabecular thickness^37^. We aimed to analyze the effects of obesity and exercise on bone mineral density and microstructure of rats through micro-CT. 

Recent studies on the correlation between obesity and bone health reported that bone mineral density decreases as the amount of body fat, blood cholesterol, neutral fat, and low-density lipoprotein cholesterol increase11, and as body mass index and waist circumference increase, leading to increased risk for osteoporosis^25^. In the present study, bone mineral density and the number of trabecular were significantly lower while trabecular thickness and gap distance were increased in rats in the high-fat diet group relative to those in rats in the normal diet group, indicating that weight gain negatively affects bone health (Fig. 2 & 3). Accumulation of adipose tissue as a result of obesity can disrupt the secretion of various substances involved in metabolism and inflammatory reactions^3^. An obesity-induced increase in the expression of regulators of inflammation such as interleukin (IL)-1, IL-6, and tumor necrosis factor-α induces osteoclast differentiation and bone resorption, and is cited as a cause of osteopenia and osteoporosis^5, 8, 10^. However, since an analysis of related factors (inflammatory cytokines, factors associated with bone resorption, etc.) was not performed in this study, it is difficult to identify methods that can confirm changes in bone mineral density and an increase in the number of trabecular due to a high-fat diet. Further investigation is necessary in this regard. 

Exercise training is commonly used to improve reduced bone mineral density, with weight training being the most common^21, 22, 27, 36^. Weight training increases mechanical load on the bones to stimulate bone metabolism and increase bone mineral density^2^. For obese patients, however, weight training can add excessive load on joints or bones, together with the increased body mass, and lead to joint injuries^19, 35^. Therefore, non-weightbearing exercises such as swimming, water walking, and cycling that are easy on joints can be suggested as an effective exercise program for obese patients with reduced bone mineral density^30^. Non-weight-bearing exercises are commonly considered inappropriate for improving bone mineral density, since they do not sufficiently stimulate the bones. However, in a comparison of the effects of swimming and jumping in animal models with reduced bone mineral density (white mice) according to the degree of hindlimb suspension, bone mineral density increased after both types of exercises^7^, suggesting that differences according to the type of exercise may be insignificant. In addition, swimming exercise followed by resistance training without a load^14^, or with a load equivalent to 16% of body weight, can effectively increase the bone mineral density in the femur and the number of trabecular that had been reduced as a result of menopause. Therefore, differences in bone mineral density according to the intensity of swimming exercise may not be significant. In this study, 8-week swimming exercise without a load improved bone mineral density that was reduced owing to obesity, increased the bone area ratio and the number of trabecular, and decreased trabecular thickness and gap distance. This finding demonstrates that although the load on the bones is reduced owing to buoyant force during swimming exercise, stimuli produced through the continuous process of contraction and relaxation of skeletal muscle reach the bones and sufficiently stimulate bone metabolism^14^. However, mechanisms by which swimming exercise promotes bone metabolism have not yet been identified in this or other studies. Yang *et al*. reported a significant increase in blood irisin concentration after 8 weeks of swimming exercise without a load^4^, and increased expression of transcription factors involved in osteoclast differentiation in primary rat osteoblasts cells and MC3T3-E1 cells treated with irisin38. Further, administration of irisin in C57BL/6 mice for 4 weeks not only increases the expression of transcription factors related to osteoclast differentiation, but also increases cortical bone in the femur and tibia6. Therefore, an increase in the level of irisin after swimming exercise may be one of the potential stimulatory mechanisms of bone metabolism. However, the roles of swimming exercise and irisin in promotion of bone metabolism have not been clearly established in previous literature or in the present study, which was aimed at analyzing the changes in bone mineral density and trabecular microstructure. Through additional research on the mechanism by which exercise training without a load improves bone mineral density, methods of improving negative effects on bones, such as reduced bone mineral density due to increased body mass, as well as safe and effective methods of exercise that can help obese patients maintain healthy bones may be established. 
